# Treatment of wounds colonized by multidrug resistant organisms in immune-compromised patients: a retrospective case series

**DOI:** 10.1186/1754-9493-7-28

**Published:** 2013-09-03

**Authors:** Marco Pignatti, Giorgio Enrico Gerunda, Gianluca Rompianesi, Nicola De Ruvo, Fabrizio Di Benedetto, Mauro Codeluppi, Decenzio Bonucchi, Lucrezia Pacchioni, Pietro Loschi, Cristina Malaventura, Giorgio De Santis

**Affiliations:** 1 Department of Plastic and Reconstructive Surgery, Policlinico of Modena,University of Modena and Reggio Emilia, Via del Pozzo 71, Modena I - 41124, Italy; 2 Liver and Multivisceral Transplant Center, University of Modena and Reggio Emilia, Via del Pozzo 71, Modena I - 41124, Italy; 3 Clinic of Infectious Diseases, Policlinico of Modena, Via del Pozzo, 71, 41124, Modena, Italy; 4 Nephrology, Dialysis and Renal Transplantation, Policlinico of Modena, Reggio Emilia, Italy; 5 Department of Medical Sciences Dept of Reproduction and Growth, Azienda Ospedaliero-Universitaria Sant’Anna Ferrara (Cona), Ferrara, Italy

**Keywords:** Abdominal wound, Wound dehiscence, Multidrug resistant organisms, Immune-compromised patients, Negative pressure therapy

## Abstract

**Background:**

Immune-compromised patients incur a high risk of surgical wound dehiscence and colonization by multidrug resistant organisms. Common treatment has been debridement and spontaneous secondary healing.

We report on the results obtained in nine such patients whose wounds were treated by debridement, negative pressure dressing and direct closure.

**Methods:**

All immune-compromised patients referred to our Institution between March 1, 2010 and November 30, 2011 for dehiscent abdominal wounds growing multidrug resistant organisms were treated by serial wound debridements and negative pressure dressing. They were primarily closed, despite positive microbiological cultures, when clinical appearance was satisfactory.

As a comparison, records from patients treated between March 1, 2008 and February 28, 2010 who, according to our Institution’s policy at that time, had been left to heal by secondary intention, were retrieved and examined.

**Results:**

Nine patients were treated by direct wound closure, five had been treated previously by secondary intention healing.

Overall, ten patients had received liver transplant, 1 kidney transplant, 1 was HIV infected, 1 suffered from multi-organ failure, 1 was undergoing hemodialysis.

Wound dehiscence involved skin and subcutaneous layers in all patients, in two the muscular layer was also involved.

Mean healing time was significantly shorter in patients treated more recently by primary intention in comparison with historical patients (28 vs 81 days). The only complication observed was a small superficial abscess that developed around a non-absorbable stitch 10 months after closure in a patient treated by primary closure.

**Conclusions:**

According to our results, fast healing can be safely obtained by closure of a clinically healthy wound, despite growth of multidrug resistant organisms, even in immune-compromised patients.

## Background

Critically ill and immune-compromised patients incur a high risk of surgical wound infection and dehiscence. The underlying disease and comorbidities such as foreign bodies (abdominal mesh, suture material), serous collections and devitalized tissues, all contribute to the risk of infection. In particular, bacterial infections contribute to the increased morbidity and mortality of patients who have undergone organ transplantation and are treated with immunosuppressive drugs and steroids [1-3].

Contamination and colonization of wounds by multidrug resistant organisms (MDRO) is becoming more frequent in the hospital setting, sometimes in the form of outbreaks, as a consequence of aggressive immunosuppressive therapies now available [4,5].

Because of the theoretical risk of systemic infection, surgical wounds contaminated or colonized by MDRO in immune-compromised patients have usually been treated by partial debridement followed by spontaneous secondary healing, although there are not, in the literature, solid data to support this practice.

On the basis of previously published data on successful closure of contaminated wounds in immune-competent patients [6,7], we hypothesized that such a procedure could be applied without danger also in the wounds contaminated or colonized by MDRO of the immune-compromised. In addition, negative pressure has been shown to be a useful and safe adjunct to treatment in difficult cases such as infected abdominal wounds, even with exposed mesh [8,9].

We report here on the results obtained in nine immune-compromised patients, whose dehiscent wounds, colonized by MDRO, were treated by direct closure. As comparison we examined the records of five patients that had been treated in the past with the more conservative approach.

## Methods

**Figure 1:**
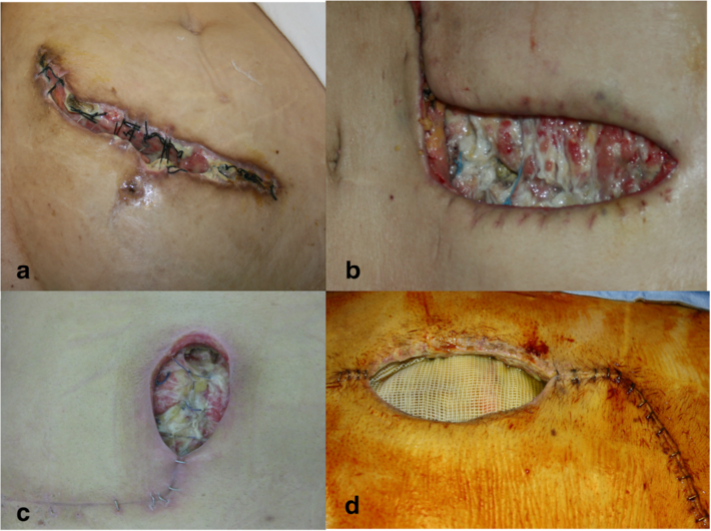
**Examples of dehiscent abdominal wounds. (a)** Dehiscent abdominal wound colonized by Acinetobacter baumannii after kidney transplantation. **(b)** Dehiscent abdominal wound colonized by Acinetobacter baumannii after liver re-transplantation (due to Hepatitis B virus). **(c)** Dehiscent abdominal wound colonized by Enterococcus faecium after liver transplantation. **(d)** Dehiscent abdominal wound colonized by Enterococcus faecium after liver transplantation. The Prolene mesh (Prolene ™. Ethicon Inc., U.S.A.), used to bridge a missing part of the muscular wall is clearly visible.

All the records of patients referred from March 1, 2008 to November 30, 2011 to the Plastic Surgery Unit of our University Hospital for the treatment of open or dehiscent abdominal wounds were reviewed (Figure 1).

Only immune-compromised patients in whom microbiological wound cultures grew MDRO are included in this report.

**Figure 2:**
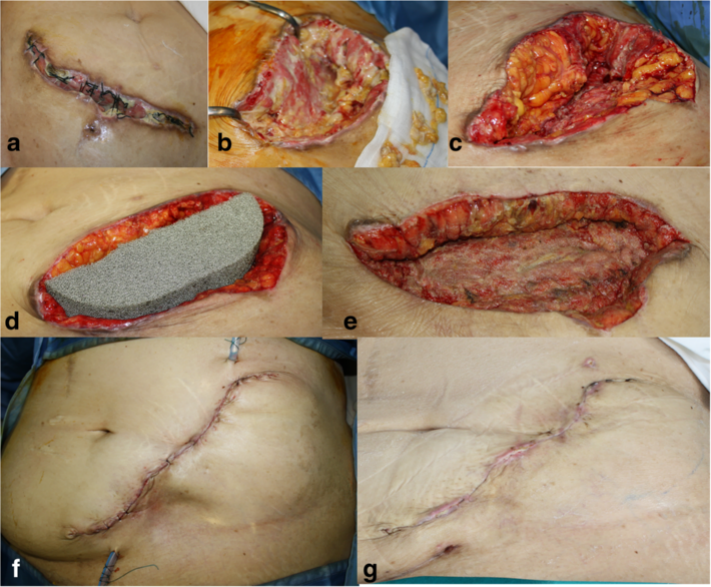
**Phases of surgical treatment.** Patient presenting with a dehiscent abdominal wound colonized by *Acinetobacter baumannii* after kidney transplant **(a)**. Serial wound debridements **(b**, **c)**. Negative pressure dressing (V.A.C. GranuFoam Silver®) positioned **(d)**. Clinically satisfactory appearance, with healthy granulating tissue despite still positive microbiological cultures **(e)**. Primary closure, 2 days post-operatively **(f)**, and 2 weeks post-operatively **(g)**.

Patients presenting on or after March 1, 2010 were treated by serial wound debridements (until healthy tissue was reached), irrigation, and negative pressure dressing (V.A.C.® Therapy, KCI UK Holdings Ltd.). Once wounds had a clinically satisfactory appearance, they were primarily closed, even in the presence of positive microbiological cultures (Figure 2).

V.A.C. was applied with a negative pressure of 125 mm/Hg, continuous mode, using the V.A.C.® GranuFoam™ dressings (black sponge), except in one case where the V.A.C. GranuFoam Silver® was used), and changed every 3–4 days, according to the usual recommendations.

Because prior to March 1, 2010, it was our Institution’s policy to treat contaminated or colonized wounds by debridement and secondary intention healing, the data of patients so treated were retrieved and used as a comparison.

Microbiological cultures of specimens collected from the wounds (swabs and tissue biopsies), were performed at first presentation and then regularly every 4 to7 days, as is the routine at our Institution, until healing was obtained.

Healing time, defined as complete skin closure, was recorded from the first diagnosis of open wound or of wound dehiscence. All wounds had healed before discharge from the hospital. After discharge, follow-up of patients of both groups was performed every two weeks for the first two months.

Approval for the retrospective review of data was obtained from the Department of Head and Neck Surgery of the University Hospital of Modena.

### Statistical analysis

Continuous data were reported as mean ± standard deviation for the parameters with a normal distribution and were compared using the two-sided Student’s *t* test or the Mann–Whitney *U* test when appropriate. Comparisons between groups for categorical variables were carried out using the χ-square test, with Yates’ correction, or Fisher’s exact test when appropriate. The p-value of 0.05 or less was considered statistically significant. All analyses were carried out using SPSS 19.0 for Windows (SPSS, Chicago, IL).

## Results

Nine patients (6 male) of a average age of 53 ± 12.2 years (range: 26–66) accessing our University Hospital on or after March 1, 2010 were treated according to the protocol mentioned above. Average number of debridements was 2.3 (range: 1 to 5).

Data from five patients treated before March 1, 2010, of an average age of 51.2 ± 8.0 years (range: 42–64) whose wound had been left to heal by secondary intention were examined for comparison.

The two series of patients were not different in terms of sex, age, etiology of immunodeficiency, size of the wounds, bacterial colonizing agents, systemic pharmacologic treatment (Table 1).

**Table 1:** Patients demographics

		**Present series**	**Historical series**
		**(9 patients)**	**(5 patients)**
**Sex (M/F)**	N	6/3	3/2
**Age (years)**	Mean ± SD	53 ± 12.2	51.2 ± 8.0
	Range	(26–66)	(42–64)
**HIV +**	N	1	1
**Transplanted**	N	6	5
**Organ failure**	N	2	0
**Wound size (cm**^**2**^**)**	Mean ± SD	51.2 ± 58.9	25 ± 9.8
	Range	(14–200)	(15–40)
**Etiological agent**	Acinetobacter baumannii	3	4
	Enterococcus faecium	3	0
	Staphylococcus aureus	1	1
	Escherichia coli	1	0
	Klebsiella	1	0

Present series: wound direct closure. Historical series: secondary intention healing.

The two groups were not statistically different in terms of sex, age, etiology of immunodeficiency, size of the wounds, bacterial colonizing agents.

The abdominal wound dehiscence involved in all cases the skin and subcutaneous layers, frequently showing an unhealthy fascio-muscular layer. In two patients the muscular layer was also open, causing continuity with the abdominal cavity. In one of the two, part of the muscular wall had been substituted with an exposed Prolene mesh (Prolene ™. Ethicon Inc., U.S.A.), while in the other patient severe edema due to an abdominal trauma and partial colectomy prevented closure.

Microbiological cultures of the wounds showed growth of multiresistant *Acinetobacter baumannii* (3 cases), *Enterococcus faecium* (3 cases), *Staphylococcus aureus* (1 case), *Escherichia coli* (1 case), *Klebsiella pneumoniae* (1 case).

In all patients, at least in one phase of the admission period, clinical signs of local infection appeared and systemic cultures (either from blood, sputum, feces, urine, mouth, skin or from all of these sites) were found to be positive for the same bacteria colonizing the wound. In 1 of the 3 patients of the study group growing Acinetobacter Baumanii in the wound, growth of the same bacteria was found in sputum and feces at the moment of wound closure and for 6 months at follow up without clinical consequences or signs of infection (coltures were then discontinued).

However, none of our patients showed clinical signs of spreading invasive infection at the moment of final closure of the wound.

Antibiotics were always used, according to the susceptibility test, for as long as the local and general condition required.

All the wounds treated by primary closure healed successfully in a average time of 28.1 ± 6.8 days (range: 18 to 30) from the beginning of treatment. In 4 of the 5 patients whose wounds that had been left to heal by secondary intention healing had been obtained in 81.2 ± 11.9 days (range: 67 to 95). The remaining patient died before the wound could heal, due to post-transplant acute hepatitis C infection.

**Figure 3:**
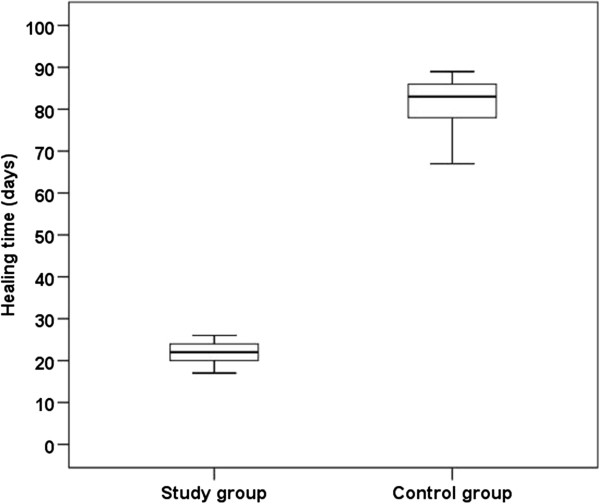
**Healing time in the case series treated by primary closure (serial debridment, negative pressure dressing, surgical closure of the wound) compared with healing time of wounds left to heal by secondary intention.** The difference is statistically significant (p = 0.002). The statistical analysis was performed with the Mann–Whitney U test.

The difference in healing time between the two series of patients was statistically significant (p = 0.002), performed with the Mann–Whitney *U* test (Figure 3). Mean follow-up was 16.1 ± 9.9 months (range: 3 to 29) for patients treated by primary closure.

The only complication observed among them was one small abscess (7 mm × 4 mm) with sinus tract that developed on a closed wound, at the cranial extremity of the xiphopubic scar, 10 months after surgery.

During surgical revision under local anesthesia, a non adsorbable stitch was found in the abscess, that grew the same *Acinetobacter baumannii* that had initially contaminated the wound, blood and airways. No further antimicrobial treatment was necessary.

In the historic series of five patients left to heal by secondary intention no local or systemic complications related to the wound treatment were reported.

## Discussion

Multidrug-resistant bacteria are becoming increasingly common as causative agents of nosocomial infections and their antimicrobial resistance is also increasing, limiting pharmacological treatment options [10,11]. *Acinetobacter baumannii, Klebsiella Pneumoniae, Staphylococcus aureus* and *Enterobacteriaceae* may cause bacteremia, pneumonia, meningitis, urinary tract and wound infection.

Risk factors for colonization or infection with multidrug-resistant species include prolonged length of hospital stay, admission to intensive care unit, mechanical ventilation, exposure to antimicrobial agents, recent surgery, invasive procedures, and severity of underlying illness [12,13]. Patients infected with these organisms are often debilitated, a fact that explains the high mortality and morbidity observed [14].

Several studies have been published on the subject of wounds contaminated or infected by MDRO, both in the population at large and in the military, whose wounds are a consequence of traumatic war injuries [14-17].

Similarly to other contaminated or infected wounds, mechanical debridement, that removes the non-viable tissue, is essential in reducing the bacterial load and eradicating the wound biofilm [18]. Leaving contaminated wounds to heal spontaneously by secondary intention has been demonstrated to be safe [7], but it requires a long healing time and frequent and time-consuming changing of wound dressings. Moreover, an open wound could become further infected and create severe problems of metabolic balance in already severely ill patients. Direct closure of such wounds presents therefore several advantages.

Although the safety of directly closing a contaminated wound has been shown in immune-competent patients [6,7,19,20], to the best of our knowledge, safety of primary MDRO-contaminated wound closure in immune-compromised patients has not yet been reported. The difference in mean healing time in patients whose wounds were closed directly (28 days) when compared with patients whose wounds in the past were left to heal by secondary intention (81 days) was clinically relevant. A shorter time of regular dressing changes means less pain and discomfort and, potentially, shorter hospitalization time.

Unfortunately, length of hospital stay and related costs of the two treatment protocols could not be compared due to multiple confounding factors. In particular, the length of hospital stay was influenced more by the underlying condition than by the delayed wound healing.

The encouraging results obtained in our (admittedly few) patients suggest that clinically healthy wounds can be closed, without severe adverse effects, also in immune-compromised patients despite the persistence of MDRO positive microbiological cultures.

However a few notes of caution are necessary. None of our patients showed signs of spreading invasive infection at the moment of final closure of the wound. In fact, while direct surgical closure can induce stable healing by primary intention in case of simple contamination (presence of non-replicating organisms), colonization (replicating microorganisms without tissue damage), or critical colonization (unhealthy granulation, no clinical signs of infection except delayed healing), surgical closure may not be safe in wounds with spreading invasive infection i.e. replicating organisms with subsequent host injury. Experienced clinical judgment of the appearance of the wound is also needed. In general, healthy, vascularised tissue should be visible on the wound surface before surgical closure is performed [21].

The role of negative pressure dressing in reducing the bacterial load while improving vascularisation and antibiotic delivery has been demonstrated [8,21].

In our patients, after the use of negative pressure dressings, improved perfusion, reduced secretions and debris of the fascial plane of the wound were observed, suggesting readiness for direct surgical closure.

A randomized study would be desirable, although critically ill, immune-compromised patients, presenting with dehiscent surgical abdominal wounds that grow MDRO are relatively rare.

In addition, given the excellent results obtained with the first few patients treated by primary closure, one wonders if leaving patients to heal by secondary intention would be ethically acceptable.

## Conclusions

Our experience, albeit small, suggests that closure of a clinically healthy wound is feasible, despite microbiological swabs positive for MDRO, even in a population of critically ill, immune-compromised patients. Serial debridement, negative pressure dressing, and direct surgical closure can lead to durable healing in a shorter time than that required by secondary intention healing.

## Abbreviations

MDRO: Multidrug resistant organisms.

## Competing interests

The authors declare that they have no competing interests.

## Authors’ contributions

MP, GR, MC Designed the study. MP, GR, MC, NDR, FDB, DB Wrote the manuscript. GR Performed the statistical analysis and prepared Figure 3. GEG, GDS Supervised the research and revised critically the manuscript. LP, PL, CM Collected and analysed the data. All authors read and approved the final manuscript.
